# Predictive value of sarcopenia-related clinical indices for early disease progression in acute pancreatitis: a multicenter retrospective cohort study

**DOI:** 10.3389/fnut.2026.1836073

**Published:** 2026-07-23

**Authors:** Xiaokai Chen, Zhengliang Li, Tao Mao, Zhitao Yang, Zongzhun Ling, Linlin Ren

**Affiliations:** 1 Department of Gastroenterology, The Affiliated Hospital of Qingdao University, Qingdao, Shandong, China; 2 Department of Cardiology, The Affiliated Hospital of Qingdao University, Qingdao, Shandong, China; 3 Department of Radiology, The Affiliated Hospital of Qingdao University, Qingdao, Shandong, China; 4 Emergency Department, Qingdao Municipal Hospital, Qingdao, Shandong, China

**Keywords:** acute pancreatitis, disease progression, predictive model, psoas muscle index, sarcopenia

## Abstract

**Background:**

Early identification of patients at risk for severe Acute Pancreatitis (AP) remains a critical clinical challenge. Although sarcopenia reflects nutritional status and physiological reserve, its predictive value for disease progression in AP remains unclear. This study aimed to evaluate the association between sarcopenia, quantified by the psoas muscle index (PMI), and progression to moderately severe or severe AP, and to develop a practical risk-stratification tool.

**Methods:**

We retrospectively analyzed 470 patients with AP. Sarcopenic status was assessed by measuring the psoas major muscle area at the L3 level on admission CT scans. Multivariate Cox regression was employed to identify independent risk factors for progression to moderately severe or severe AP (MSAP/SAP). A predictive nomogram was constructed and validated using C-index, time-dependent ROC curves, and decision curve analysis (DCA).

**Results:**

Decreased PMI and Albumin (Alb), alongside elevated Systemic Immune-Inflammation index (SII) and Alanine Aminotransferase (ALT), were independent predictors of disease progression. The nomogram demonstrated moderate discriminatory ability, with C-indices of 0.68 and 0.63 in the training and validation sets, respectively. The 7-day Area Under the Curve (AUC) reached 0.69 (training) and 0.65 (validation). Survival analysis demonstrated significantly higher progression rates in the high-risk group (*P* < 0.001), and DCA indicated a meaningful clinical net benefit.

**Conclusion:**

Sarcopenia, characterized by a low PMI, is an independent predictor of early progression to moderately severe or severe AP. The developed nomogram, integrating PMI with routine laboratory markers (SII, ALT, Alb), may serve as a potentially useful tool for early risk stratification and personalized management.

## 1 Introduction

Acute Pancreatitis (AP) is one of the most common gastrointestinal emergencies worldwide, and imposes a considerable burden on healthcare systems (1, 2). Although most cases are mild and self-limiting, approximately 15%–20% of patients develop Moderately Severe Acute Pancreatitis (MSAP) or Severe Acute Pancreatitis (SAP) (3). SAP is characterized by pancreatic necrosis and persistent organ failure and is associated with a mortality rate of 20%–30% (4). Because clinical deterioration may occur rapidly, early identification of patients at high risk for progression to severe forms is essential for timely intervention and improved outcomes.

Several clinical scoring systems, including APACHE II, Ranson, and BISAP, are widely used to stratify disease severity. However, their clinical utility is often limited by complexity and delayed availability, as many variables require 24–48 h to obtain, precluding timely intervention during the critical window for early management (5). These limitations underscore the need for objective, stable, and readily accessible indicators that can be assessed at admission to improve early risk stratification.

Sarcopenia, defined as a progressive loss of skeletal muscle mass and function, has emerged as an important prognostic factor in various acute and chronic conditions (6, 7). Beyond its traditional association with aging, sarcopenia is increasingly recognized as a marker of reduced physiological reserve and systemic vulnerability (8). In gastrointestinal diseases, its adverse prognostic impact has been well established in liver cirrhosis and malignancies (9–11). However, its role in acute inflammatory disorders such as AP remains incompletely understood and somewhat controversial. While some studies suggest that muscle depletion reflects diminished resilience to systemic inflammatory stress, others have reported inconsistent associations with clinical outcomes. It has been hypothesized that a low-reserve state, reflected by reduced muscle mass, may impair the host response to the Systemic Inflammatory Response Syndrome (SIRS) characteristic of AP, thereby increasing the risk of early disease progression (12–14).

Although dual-energy X-ray absorptiometry (DXA) and bioelectrical impedance analysis (BIA) are the most commonly employed methods for body composition assessment, their utility in emergency settings remains limited. Conversely, contrast-enhanced Computed Tomography (CT) scans are routinely performed in patients with AP for diagnostic evaluation and staging (15). CT-derived parameters, such as the Psoas Muscle Index (PMI), enable opportunistic and quantitative assessment of muscle mass without additional cost or radiation exposure (16). However, whether CT-assessed sarcopenia can enhance early risk prediction in AP has not been fully elucidated.

Therefore, this study aimed to investigate the association between admission CT-assessed sarcopenia and progression to MSAP/SAP. We further sought to integrate PMI with routine laboratory markers to construct and validate a predictive nomogram for individualized early risk stratification.

## 2 Materials and methods

### 2.1 Study design and population

**FIGURE 1:**
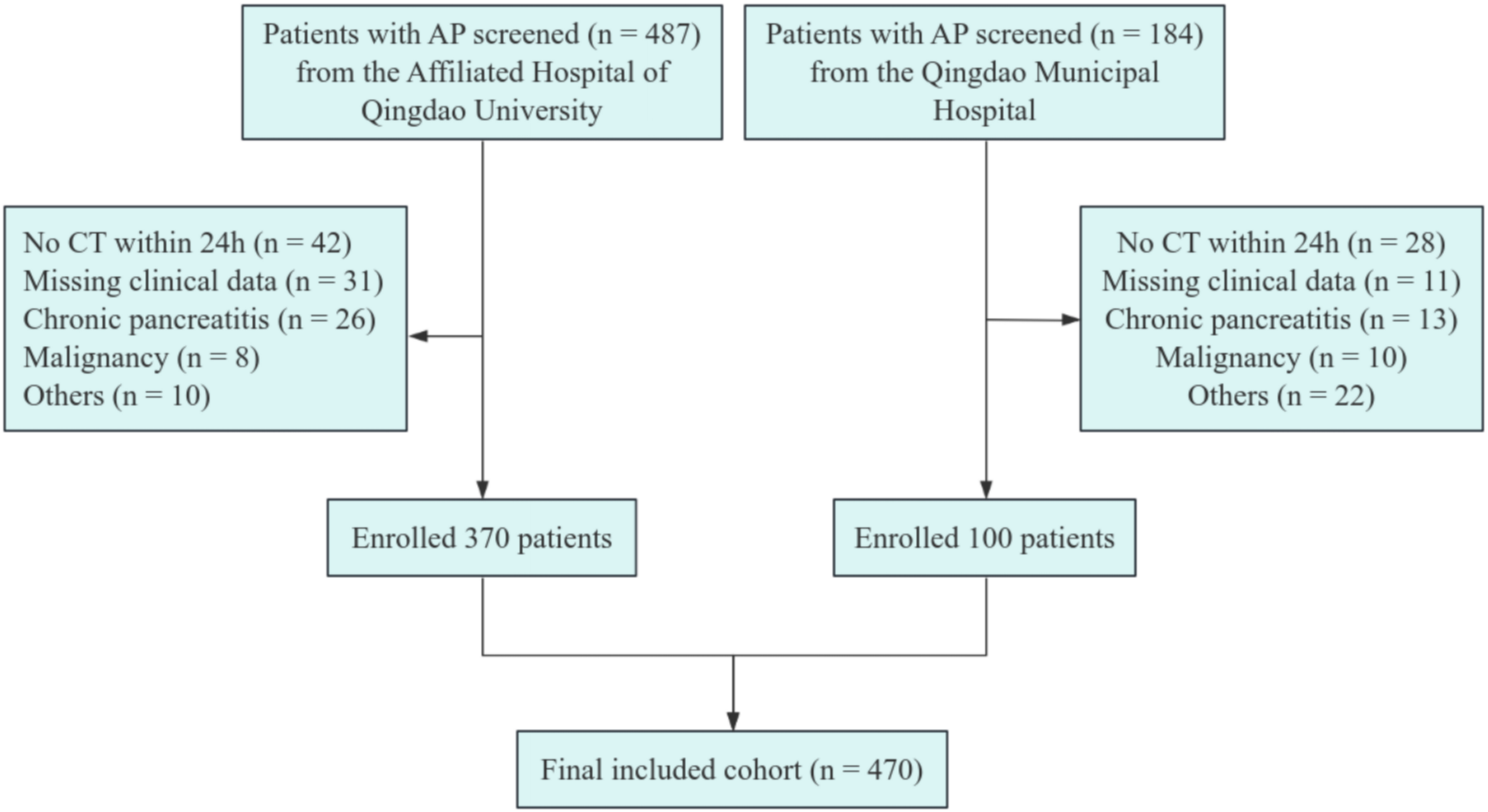
Flow chart of study population screening.

This multicenter retrospective cohort study included 470 patients diagnosed with AP between January 2019 and December 2024 at the Affiliated Hospital of Qingdao University (*n* = 370) and Qingdao Municipal Hospital (*n* = 100). The study protocol was approved by the Institutional Review Board of the participating centers, and informed consent was waived due to the retrospective nature of the study. Inclusion criteria were: (1) age ≥18 years; (2) a confirmed diagnosis of Mild Acute Pancreatitis (MAP) upon admission according to the Revised Atlanta Classification; (3) available abdominal CT images and complete clinical records within 24 h of admission. Exclusion criteria included: (1) pregnancy; (2) AP secondary to endoscopic retrograde cholangiopancreatography (ERCP) or pancreatic surgery; (3) chronic pancreatitis; (4) periampullary or other malignancies; (5) pre-existing chronic wasting diseases or disorders affecting skeletal muscle metabolism. The patient selection process, including the number of screened patients, exclusions, and reasons for exclusion, is presented in Figure 1.

### 2.2 Data collection and variable definitions

Comprehensive clinical data were extracted from electronic medical record systems, including demographics, comorbidities, and laboratory parameters obtained upon admission. Several indices were calculated as follows: Neutrophil-to-Lymphocyte Ratio (NLR) = Neutrophil count/Lymphocyte count. Systemic Immune-Inflammation Index (SII) = Platelet count × Neutrophil count/Lymphocyte count.

### 2.3 Radiologic assessment of sarcopenia

**FIGURE 2:**
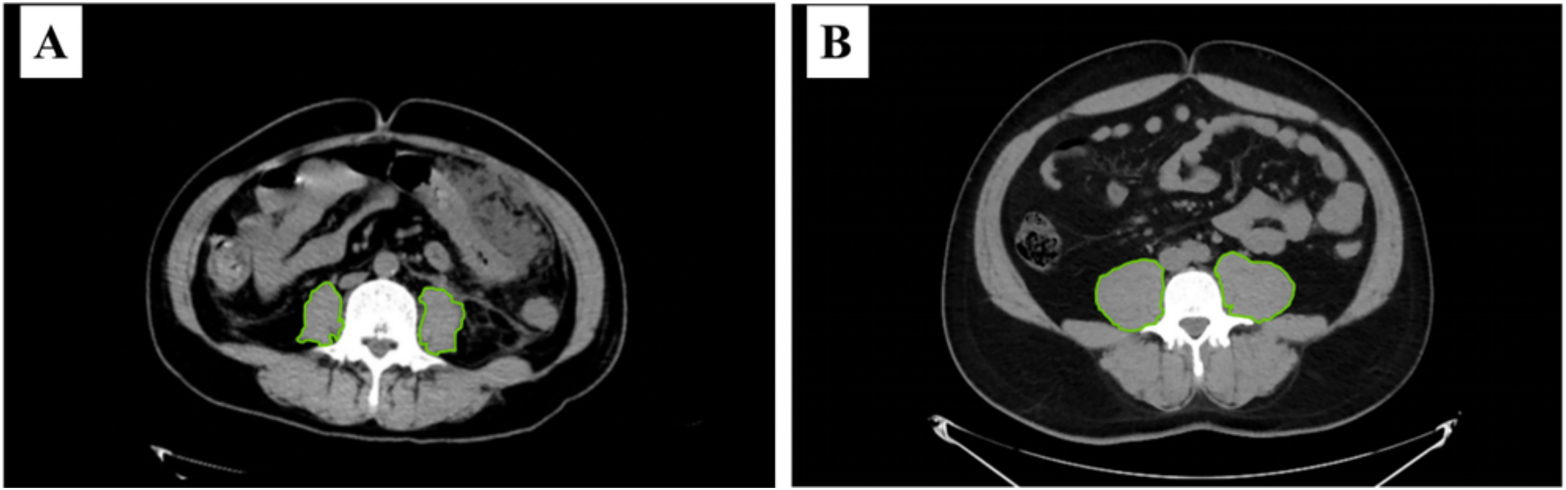
PMA measured on CT images of different patients. Representative axial CT images showing the psoas muscle (outlined in green) at the level of the third lumbar vertebra from a patient with low PMA (**A**) and a patient with high PMA (**B**).

Abdominal CT images (in DICOM format) obtained within 24 h of admission were retrieved from the Picture Archiving and Communication System (PACS) at two participating hospitals. All scans were contrast-enhanced CT in the portal venous phase, with a standardized imaging protocol and fixed slice thickness of 2–3 mm applied consistently across both centers. Skeletal muscle parameters were analyzed at the third lumbar vertebra (L3) level using Siemens syngo.via workstation. Two radiologists, blinded to the clinical outcomes, independently measured the Psoas Major Muscle Area (PMA) using a threshold of −29 to +150 Hounsfield units (HU). Standardized measurement criteria were applied to ensure reliability, and the average of the two measurements was used for analysis. The PMI was then calculated to adjust for body habitus: PMI (cm^2^/m^2^) = PMA (cm^2^)/Height (m^2^) (17) (Figure 2). According to the imaging evaluation and diagnostic criteria adopted in previous Chinese multicenter studies and studies from several other Asian countries (18–20), sarcopenia was defined as L3-PMI <5.41 cm^2^/m^2^ in male patients and L3-PMI <3.32 cm^2^/m^2^ in female patients.

### 2.4 Outcome measures and follow-up

The primary outcome was progression from initially MAP to MSAP/SAP during hospitalization, defined according to the Revised Atlanta Classification.

MSAP was defined as the presence of transient organ failure (resolving within 48 h) and/or local or systemic complications without persistent organ failure. SAP was defined as persistent organ failure lasting ≥48 h, assessed using the modified Marshall scoring system.

Secondary outcomes included local complications (e.g., infected necrosis), worsening systemic inflammation (defined as C- Reactive Protein (CRP) > 150 mg/L at 72–96 h post-admission), ICU admission, and hospital length of stay.

Follow-up commenced at admission and continued until the occurrence of the primary outcome (i.e., progression to MSAP or SAP) or hospital discharge, whichever came first. The time-to-event was defined as the interval from admission to the first occurrence of MSAP or SAP.

### 2.5 Statistical analysis and model development

All included patients had complete baseline data, no imputation methods were applied. Statistical analyses were performed using R (v4.3.3) and IBM SPSS Statistics (v27.0). Continuous variables are presented as mean ± standard deviation (SD) or median (interquartile range, IQR), and categorical variables as frequencies (*n*, %). Groups were compared using the Student’s *t*-test, Mann–Whitney *U* test, or χ^2^ test as appropriate. Data from the two participating centers were pooled, and patients were randomly assigned to training and validation cohorts.

Given that the primary outcome was time to progression from admission to moderately severe or severe acute pancreatitis (MSAP/SAP), Cox proportional hazards regression was applied to account for both the occurrence and timing of events. Patients who did not experience progression were treated as censored at the time of hospital discharge. To mitigate multicollinearity, variables with high biological or statistical correlation (e.g., PMA and PMI) were handled by retaining only one variable (PMI) based on clinical relevance (better normalization for body size). Independent risk factors identified via multivariate Cox regression were used to construct a predictive nomogram. For variables without established clinical thresholds (e.g., SII), optimal cut-off values were determined using the Youden index from ROC curve analysis, while other variables were categorized based on clinically recognized reference values.

The model’s performance was evaluated using the Concordance index (C-index), time-dependent ROC curves, and calibration curves. Clinical utility was assessed using Decision Curve Analysis (DCA). The proportional hazards assumption was assessed using Schoenfeld residuals, and no significant violations were observed. The 7-day time point was selected for clinically relevant early risk stratification, while the Cox model utilized the full time-to-event information during hospitalization. Survival curves for high- and low-risk groups (stratified by nomogram-predicted risk) were generated via the Kaplan–Meier method and compared using the log-rank test. A two-tailed *P*-value < 0.05 was considered statistically significant.

## 3 Results

### 3.1 Baseline characteristics and clinical outcomes of the study population

The total cohort comprised 470 patients, who were randomly assigned to a training set (*n* = 329) and a validation set (*n* = 141) at a 7:3 ratio. To ensure a balanced representation of patients from both centers, stratified random sampling was performed based on the study site. As shown in Table 1, baseline demographic and clinical characteristics were generally comparable between the two cohorts. The proportion of male patients was 62.92% in the training set and 67.38% in the validation set (*P* = 0.355). The prevalence of sarcopenia did not differ significantly between the two sets (25.53% vs. 21.28%, *P* > 0.05). With the exception of hypertension prevalence (*P* = 0.034), no other clinical variables – including etiology and laboratory parameters – differed significantly between the cohorts (all *P* > 0.05), indicating acceptable comparability for subsequent model development and validation.

**TABLE 1:** Comparison of baseline data between training set and validation set (*N*, %).

Variables	Training set (*n* = 329)	Validation set (*n* = 141)	*P*
Gender			0.355
Female	122 (37.08)	46 (32.62)	
Male	207 (62.92)	95 (67.38)	
Age, years			0.596
<45	131 (39.82)	60 (42.55)	
45–65	109 (33.13)	40 (28.37)	
>65	89 (27.05)	41 (29.08)	
BMI, kg/m^2^			0.105
<18	8 (2.43)	3 (2.13)	
18–24	104 (31.61)	29 (20.57)	
24–28	123 (37.39)	61 (43.26)	
>28	94 (28.57)	48 (34.04)	
Hypertension			0.034
No	227 (69.00)	83 (58.87)	
Yes	102 (31.00)	58 (41.13)	
Diabetes			0.639
No	238 (72.34)	99 (70.21)	
Yes	91 (27.66)	42 (29.79)	
Etiology			0.536
Gallstone-related	132 (40.12)	50 (35.46)	
Alcohol-related	143 (43.47)	69 (48.94)	
Hyperlipidemia-related	54 (16.41)	22 (15.60)	
Smoke			0.242
No	237 (72.04)	94 (66.67)	
Yes	92 (27.96)	47 (33.33)	
Drink			0.302
No	235 (71.43)	94 (66.67)	
Yes	94 (28.57)	47 (33.33)	
Sarcopenia			0.571
No	245 (74.47)	111 (78.72)	
Yes	84 (25.53)	30 (21.28)	
PMA, cm^2^	5.02 (3.76, 6.79)	4.93 (3.84, 6.43)	0.939
PMI, cm^2^/m^2^	5.08 (4.20–6.78)	4.86 (3.64–6.75)	0.160
PMD, HU	42.75 (35.38-46.50)	41.50 (34.50–46.50)	0.960
Erythrocyte, 10^9^/L	4.48 (3.90, 5.16)	4.56 (3.93, 5.14)	0.825
Leukocytes, 10^9^/L	12.09 (8.20, 15.93)	12.60 (8.25, 16.73)	0.585
Neutrophils, 10^9^/L	9.92 (6.31, 13.76)	10.62 (6.46, 15.06)	0.270
Platelets, 10^9^/L	214.00 (173.00, 271.00)	201.00 (166.00, 270.00)	0.211
Lymphocytes, 10^9^/L	1.14 (0.83, 1.62)	1.12 (0.76, 1.69)	0.580
Hematocrit, %	41.30 (36.00, 46.20)	42.20 (35.80, 46.20)	0.843
PCT, ng/ml	0.59 (0.10, 1.82)	0.51 (0.12, 2.26)	0.952
CRP, mg/L	74.40 (10.16, 160.40)	84.84 (20.85, 154.87)	0.678
NLR	8.37 (4.65, 14.39)	9.01 (4.75, 14.97)	0.403
SII	1774.65 (890.06, 3139.47)	1946.05 (980.76, 3150.43)	0.536
ALT, U/L	35.24 (21.10, 57.39)	31.30 (22.00, 49.10)	0.344
AST, U/L	37.10 (21.00–62.62)	31.90 (20.33–60.25)	0.350
Alb, g/L	35.60 (30.30, 41.41)	35.20 (29.50, 39.00)	0.263
Urea nitrogen, mmol/L	5.60 (3.90, 7.50)	5.30 (4.10, 8.10)	0.711
Creatinine, μmol/L	67.20 (53.10, 90.30)	65.50 (51.30, 89.00)	0.750
Total bilirubin, μmol/L	18.10 (13.57–26.42)	17.90 (12.13–24.55)	0.450
Glucose, mmol/L	9.55 (7.10, 13.60)	9.90 (7.22, 13.49)	0.705
Serum calcium, mmol/L			0.274
2.10–2.55	151 (45.90)	57 (40.43)	
<2.10, >2.55	178 (54.10)	84 (59.57)	
Serum sodium, mmol/L			0.586
135–145	225 (68.39)	100 (70.92)	
<135, >145	104 (31.61)	41 (29.08)	

A detailed comparison of baseline characteristics between patients with and without sarcopenia is provided in Supplementary Appendix A.1. Briefly, patients in the sarcopenia group were significantly older and had a higher prevalence of biliary-origin AP compared to the non-sarcopenia group (*P* < 0.05). Notably, the incidence of early disease progression was significantly higher in the sarcopenia group than in the non-sarcopenia group (47.37% vs. 33.43%, *P* = 0.01). This preliminary association between baseline muscle depletion and clinical deterioration provided the rationale for further investigating its independent prognostic value in the subsequent regression analyses.

### 3.2 Screening for independent predictors of early disease progression in AP

To identify potential predictors of progression with AP, univariate Cox proportional hazards regression analysis was initially performed on all baseline clinical and laboratory parameters. As shown in Table 2, several demographic factors were significantly associated with an increased risk of early disease progression, including a history of hypertension, advanced age, and lower BMI (all *P* < 0.05).

**TABLE 2:** Univariate Cox regression and multivariate COX regression analysis.

	Univariate Cox analysis					Multivariate Cox analysis				
Variables	β	S.E	Z	*P*	HR (95% CI)	β	S.E	Z	*P*	HR (95% CI)
Gender										
Female					1.00 (Reference)					1.00 (Reference)
Male	−0.31	0.15	−2.03	0.042	0.73 (0.55–0.99)	−0.16	0.18	−0.89	0.374	0.85 (0.60–1.21)
Age, years										
<45					1.00 (Reference)					1.00 (Reference)
45–65	0.42	0.18	2.36	0.018	1.53 (1.07–2.17)	0.09	0.20	0.46	0.647	1.10 (0.74–1.63)
>65	0.46	0.19	2.45	0.014	1.59 (1.10–2.30)	−0.00	0.24	−0.00	1.000	1.00 (0.62–1.60)
BMI, kg/m2										
<18	0.33	0.43	0.76	0.450	1.38 (0.60–3.22)					
18–24					1.00 (Reference)					
24–28	−0.22	0.18	−1.21	0.228	0.80 (0.56–1.15)					
>28	0.02	0.19	0.13	0.900	1.02 (0.71–1.48)					
Hypertension										
No					1.00 (Reference)					1.00 (Reference)
Yes	0.34	0.16	2.19	0.028	1.40 (1.04–1.90)	0.22	0.18	1.22	0.223	1.24 (0.88–1.77)
Diabetes										
No					1.00 (Reference)					
Yes	0.06	0.17	0.37	0.715	1.06 (0.77–1.47)					
Etiology										
Gallstone-related					1.00 (Reference)					
Alcohol-related	−0.06	0.16	−0.38	0.702	0.94 (0.69–1.29)					
Hyperlipidemia-related	−0.24	0.23	−1.04	0.300	0.79 (0.50–1.24)					
Smoke										
No					1.00 (Reference)					
Yes	0.00	0.17	0.03	0.979	1.00 (0.73–1.39)					
Drink										
No					1.00 (Reference)					
Yes	−0.09	0.17	−0.56	0.574	0.91 (0.66–1.26)					
PMI, cm^2^/m^2^	−0.14	0.04	−3.97	<0.01	0.87 (0.81–0.93)	−0.13	0.03	−4.33	<0.001	0.88 (0.83–0.94)
Erythrocyte, 10^9^/L	−0.00	0.04	−0.05	0.964	1.00 (0.92–1.08)					
Leukocytes, 10^9^/L	−0.00	0.01	−0.17	0.862	1.00 (0.97–1.02)					
Neutrophils, 10^9^/L	−0.01	0.01	−1.00	0.203	0.99 (0.97–1.01)					
Platelets, 10^9^/L	−0.01	0.01	−0.82	0.414	0.99 (0.97–1.01)					
Lymphocytes, 10^9^/L	−0.00	0.00	−1.48	0.139	1.00 (0.99–1.01)					
Hematocrit, %	0.06	0.09	0.67	0.500	1.07 (0.89–1.28)					
PCT, ng/ml	0.01	0.00	2.49	0.013	1.01 (1.01–1.02)					
CRP, mg/L	−0.00	0.00	−0.12	0.902	1.00 (0.99–1.01)					
SII, ×109/L										
300–600					1.00 (Reference)					1.00 (Reference)
>600	0.49	0.20	2.51	0.012	1.64 (1.11–2.41)	0.56	0.21	2.71	0.007	1.75 (1.17–2.62)
ALT, U/L										
<40					1.00 (Reference)					1.00 (Reference)
41–140	0.23	0.17	1.37	0.172	1.26 (0.91–1.74)	0.11	0.19	0.61	0.545	1.12 (0.78–1.61)
>140	1.07	0.21	5.00	<0.001	2.92 (1.92–4.44)	0.92	0.24	3.76	<0.001	2.50 (1.55–4.03)
Alb, g/L										
<25	0.67	0.31	2.16	0.030	1.96 (1.07–3.59)	0.61	0.35	1.75	0.050	1.84 (1.63–3.66)
25–30	0.17	0.21	0.84	0.401	1.19 (0.79–1.78)	0.27	0.23	1.19	0.235	1.31 (0.84–2.04)
30–35	0.23	0.18	1.30	0.195	1.26 (0.89–1.78)	0.32	0.20	1.65	0.100	1.38 (0.94–2.02)
>35					1.00 (Reference)					1.00 (Reference)
Urea nitrogen, mmol/L										
<8					1.00 (Reference)					1.00 (Reference)
8–14.3	0.38	0.21	1.81	0.070	1.46 (0.97–2.19)	0.28	0.23	1.20	0.232	1.32 (0.84–2.07)
>14.3	0.53	0.22	2.44	0.015	1.70 (1.11–2.59)	0.21	0.30	0.69	0.493	1.23 (0.68–2.22)
Creatinine, μmol/L										
44–133					1.00 (Reference)					1.00 (Reference)
>133	0.30	0.16	1.85	0.064	1.36 (0.98–1.87)	0.08	0.23	0.33	0.741	1.08 (0.68–1.71)
Total bilirubin, μmol/L	0.01	0.03	2.87	0.004	1.01 (1.00–1.02)	0.25	0.17	1.47	0.141	1.28 (0.92–1.79)
Glucose, mmol/L	−0.02	0.01	−1.40	0.162	0.98 (0.95–1.01)
serum calcium, mmol/L										
2.10–2.55					1.00 (Reference)					
<2.10, >2.55	0.02	0.15	0.12	0.908	1.02 (0.75–1.37)					
serum sodium, mmol/L										
135–145					1.00 (Reference)					
<135, >145	0.01	0.16	0.09	0.926	1.01 (0.74–1.39)					

HR, Hazard ratio. Selected continuous variables were categorized based on clinical thresholds or optimal cut-off values to enhance model interpretability and address minimal unit effect sizes in the initial Cox proportional hazards analysis.

Regarding laboratory and clinical indices, hypocalcemia, lower PMI, elevated BUN, and increased serum creatinine were significant predictors of early disease progression. Patients were stratified according to SII levels. The optimal cut-off value for SII, determined using the Youden index, was 600 × 10^9^/L. Furthermore, higher levels of SII, total bilirubin, ALT, Procalcitonin (PCT), and fasting blood glucose were associated with an elevated risk of clinical deterioration. Conversely, higher Alb levels were significantly associated with a reduced risk of early disease progression (all *P* < 0.05).

Candidate variables with statistical significance in the univariate analysis were subsequently entered into a multivariate Cox proportional hazards regression model to identify independent risk factors (Table 2). After adjusting for potential confounding variables, PMI, SII, Alb, and ALT remained independent predictors of disease progression in AP patients (all *P* < 0.05). Specifically, lower PMI was identified as a robust independent predictor of early clinical deterioration. In contrast, elevated SII, increased ALT, and hypoalbuminemia significantly increased the risk of disease progression.

Based on these multivariate regression results, PMI, SII, ALT, and Alb were selected as the final predictors to construct a clinical nomogram, which was developed to quantitatively predict the risk of 7-day progression to MSAP or SAP in hospitalized AP patients.

### 3.3 Development and performance evaluation of the predictive nomogram

**FIGURE 3:**
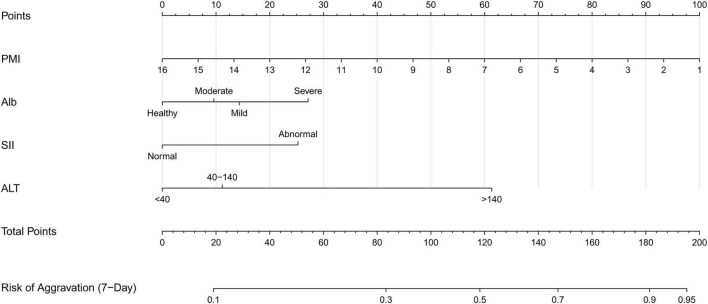
Nomogram prediction model for 7-day disease progression in patients with AP. The nomogram incorporates independent prognostic factors to estimate the individual risk of early disease progression.

Based on the independent predictors identified in the multivariate Cox regression analysis (PMI, ALT, Alb, and SII), a nomogram was constructed to estimate the probability of progression to MSAP or SAP within 7 days (Figure 3). Each variable was assigned a weighted score proportional to its regression coefficient, and the total score corresponded to an individualized predicted risk of early progression.

#### 3.3.1 Model discrimination and calibration

**FIGURE 4:**
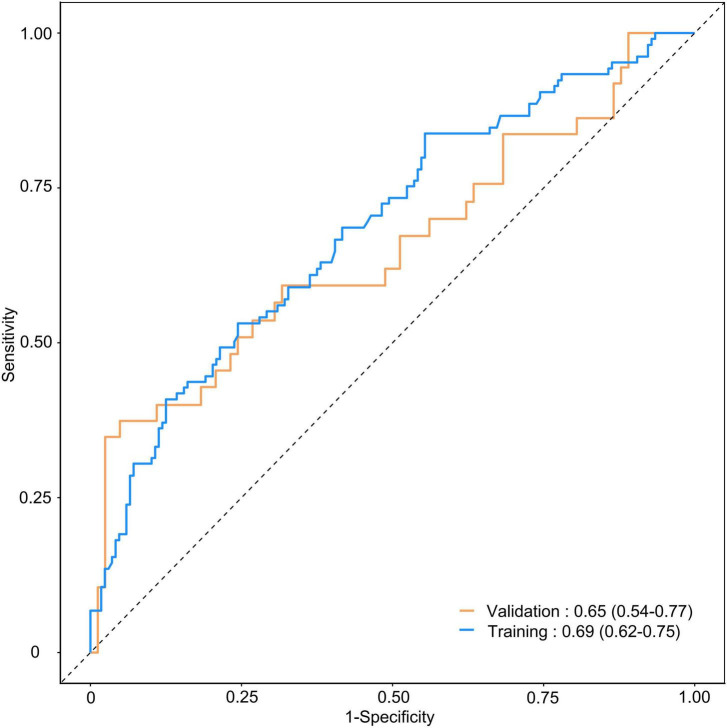
ROC curves of the nomogram model in the training and validation sets. The AUC with 95% CI is shown for each set.

The predictive performance of the nomogram was evaluated in both the training and validation cohorts. The concordance index (C-index) was 0.68 in the training cohort and 0.63 in the validation cohort. Time-dependent receiver operating characteristic (ROC) curve analysis at 7 days demonstrated an area under the curve (AUC) of 0.69 (95% CI: 0.62–0.75) in the training cohort and 0.65 (95% CI: 0.54–0.77) in the validation cohort (Figure 4).

**FIGURE 5:**
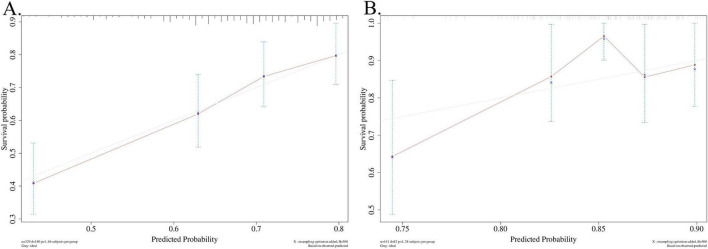
Calibration curves of the nomogram model in the training set (**A**) and validation set (**B**). The *x*-axis represents predicted risk probability, and the *y*-axis represents actual observed probability. The diagonal line indicates ideal consistency between prediction and observation. Calibration was evaluated using 500 bootstrap resamples.

Calibration curves were generated to assess the agreement between predicted probabilities and observed outcomes (Figure 5). The calibration plots showed good agreement between predicted and observed risks in both cohorts.

#### 3.3.2 Clinical utility analysis

**FIGURE 6:**
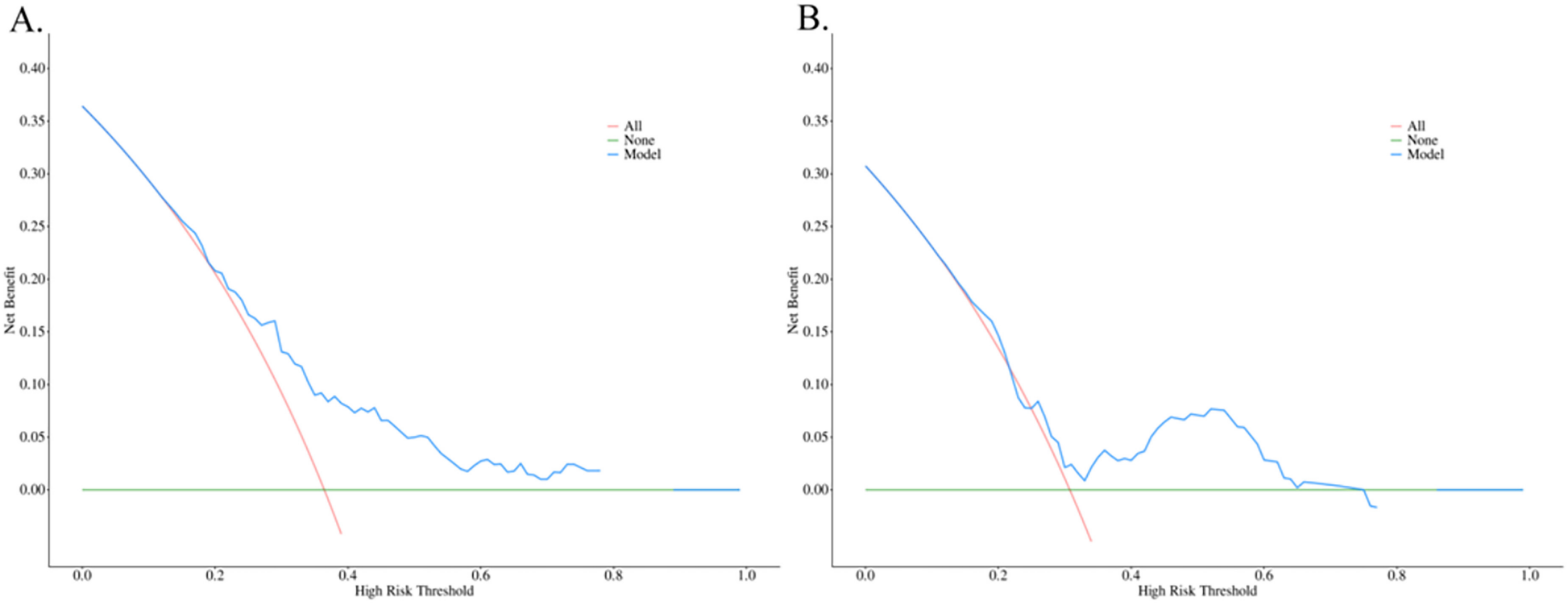
DCA of the nomogram model in the training set (**A**) and validation set (**B**). The *x*-axis is the high-risk threshold probability, and the *y*-axis is the net benefit of using the model.

Decision curve analysis (DCA) was performed to evaluate the clinical usefulness of the nomogram (Figure 6). Across a range of threshold probabilities (0.0–0.8), the nomogram demonstrated a higher net benefit compared with the treat-all and treat-none strategies.

### 3.4 Clinical utility and risk stratification analysis

**FIGURE 7:**
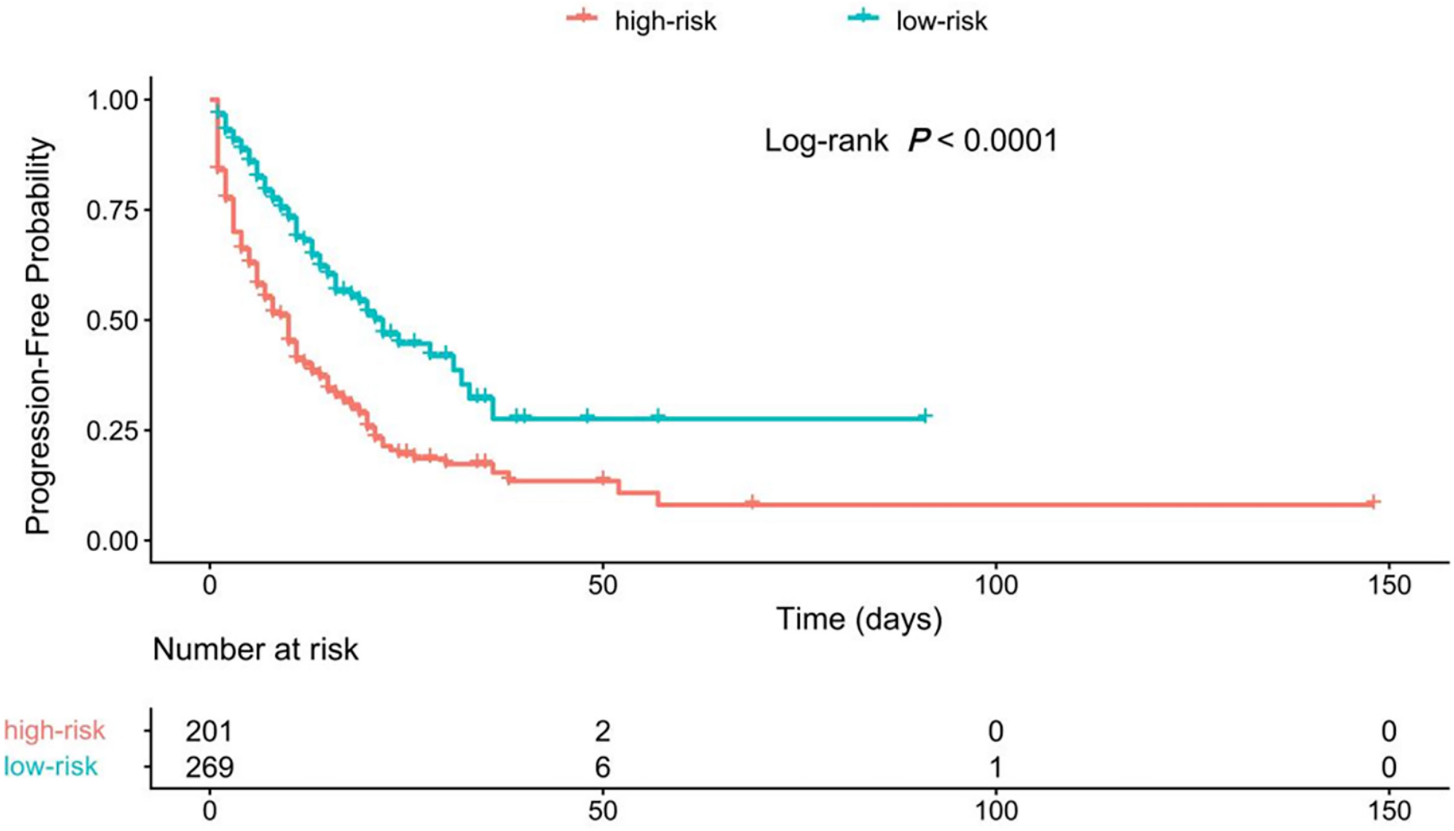
Progression-free survival curves of the high-risk group and the low-risk group. Patients were stratified into high-risk and low-risk groups based on the nomogram’s predicted risk of 7-day progression. Progression-free survival curves were compared using the log-rank test. The number of patients at risk in each group at different time points is shown below the curves.

To assess the prognostic stratification ability of the nomogram, the total cohort (*n* = 470) was categorized into a high-risk group (*n* = 201) and a low-risk group (*n* = 269) according to the optimal risk score cut-off derived from the Youden index. Time-to-event was defined as the interval from hospital admission to early disease progression, consistent with the definition described in the Methods section. Kaplan–Meier analysis was performed to compare progression-free survival between the two groups (Figure 7 and Table 3).

**TABLE 3:** Comparison of early disease progression among patients in different risk groups.

Group	Total (*n*)	Progressing cases (*n*)	Median time to progression (days, 95% CI)	Log-rank test (χ^2^/*P*)
High-risk	201	182	10 (7–11)	18.75/ *P* < 0.001
Low-risk	269	80	22 (16–33)	

A significant difference in progression-free survival was observed between the high-risk and low-risk groups. In the high-risk group, 182 patients experienced early disease progression, with a median time to progression of 10 days (95% CI: 7–11 days). In the low-risk group, 80 patients developed progression, with a median time to progression of 22 days (95% CI: 16–33 days). The log-rank test demonstrated a statistically significant difference between the two groups (χ^2^ = 18.75, *P* < 0.0001).

## 4 Discussion

This retrospective cohort study evaluated whether CT-derived sarcopenia parameters, in combination with inflammatory and nutritional markers, could predict early disease progression in AP. Our findings suggest that reduced physiological reserve and heightened inflammatory activation are associated with early clinical deterioration. Specifically, a low PMI, hypoalbuminemia, an elevated SII, and increased ALT independently predicted progression from mild to moderate/severe AP. The nomogram integrating these variables demonstrated moderate discrimination alongside satisfactory calibration within the key 7-day post-admission period, supporting its potential role as a preliminary risk stratification tool.

Sarcopenia has increasingly been recognized as an alternative marker of diminished physiological reserve and metabolic vulnerability (7, 21). In the present study, PMI was quantified at the L3 level using admission CT scans, enabling an objective, opportunistic assessment without the need for additional procedures. We observed that a lower PMI was significantly associated with early disease progression. This finding expands upon prior research – which has primarily linked sarcopenia to adverse outcomes in conditions like chronic pancreatitis, chronic liver disease, malignancy, or studies of long-term mortality (10, 22, 23) – by demonstrating its prognostic relevance in the acute inflammatory setting of AP. Mechanistically, skeletal muscle represents the body’s largest protein reservoir and plays a central role in metabolic adaptation during stress. In the hypermetabolic state of AP, reduced muscle mass may reflect critically impaired metabolic resilience. Furthermore, skeletal muscle also functions as an endocrine organ, secreting myokines that regulate systemic inflammation and immune homeostasis (24). In individuals with muscle depletion, an exaggerated systemic inflammatory response and immune dysregulation may not be adequately counterbalanced, thereby facilitating organ dysfunction and accelerating disease progression (14). Our findings support the hypothesis that CT-assessed muscle depletion is particularly informative during the early critical window of early disease progression.

The SII, which integrates neutrophil, lymphocyte, and platelet counts, reflects the balance between inflammatory activation and immune suppression (25). Consistent with established pathophysiological models of AP, we found that an elevated SII was a strong independent predictor of progression [HR: 1.75 (1.17–2.62)], underscoring the central role of SIRS and immune imbalance in early organ dysfunction. Although ALT is not traditionally incorporated into conventional AP severity scores, its independent association with early disease progression in our cohort may indicate concomitant hepatocellular stress, a biliary etiology, or microcirculatory disturbances during the early phase of inflammation (26–28). Similarly, albumin, a classical marker of nutritional status and systemic capillary leakage, contributes biological plausibility to the predictive model, as hypoalbuminemia reflects both baseline vulnerability and inflammation-induced fluid redistribution.

Notably, serum calcium, a conventional prognostic marker in acute pancreatitis (29), did not demonstrate independent predictive value in our model. This observation may reflect the time-dependent nature of calcium alterations during the course of AP. In the very early phase of the disease (within 24 h of admission), serum calcium levels may remain relatively preserved due to physiological compensatory mechanisms and the delayed development of extensive fat necrosis. In contrast, persistent hypocalcemia is more commonly observed during later stages of disease progression and has been associated with more severe inflammatory injury (30).

AP typically follows a dynamic and biphasic clinical course, with early deterioration driven predominantly by systemic inflammation and organ failure (31). We therefore selected a 7-day predictive window to capture the period of most clinically significant progression. This timeframe aligns with current clinical practice, where early aggressive management – such as optimized fluid resuscitation, close hemodynamic monitoring, and the timely initiation of enteral nutrition – can fundamentally alter the disease trajectory. The identification of high-risk patients upon admission could thus facilitate proactive management strategies rather than reactive escalation after organ dysfunction has already developed. Although the model demonstrated moderate discriminative performance (AUC 0.65–0.69), this is comparable to previously reported early prediction models in acute pancreatitis. The moderate performance may reflect the inherent complexity and dynamic nature of disease progression in AP, particularly when predictions are based on baseline variables alone. In this context, our nomogram offers a practical and cost-effective approach, integrating routinely available imaging and laboratory parameters into a visually intuitive decision-support tool.

Several limitations warrant consideration. First, the retrospective design may introduce selection bias and residual confounding. Second, although multicenter data were utilized, the unbalanced sample sizes between institutions necessitated pooling the data for a stratified random split rather than performing a site-specific external validation, therefore, further validation in larger, more ethnically diverse cohorts is essential to confirm generalizability. The lack of true external validation represents a major limitation that may restrict the generalizability of our findings. Third, we did not perform direct comparisons with established clinical severity scoring systems (e.g., APACHE II, Ranson, or BISAP) within the same cohort, which may limit the ability to contextualize the relative performance of our model. Lastly, our assessment focused solely on muscle cross-sectional area; future investigations incorporating functional measures of muscle strength and performance may provide a more comprehensive characterization of sarcopenic status. Prospective studies are ultimately needed to determine whether intervention strategies guided by early sarcopenia-based risk stratification can significantly improve clinical outcomes.

## 5 Conclusion

This study demonstrates that CT-derived sarcopenia (PMI) is an independent predictor of early progression in acute pancreatitis. Our nomogram demonstrates moderate predictive performance and may serve as a preliminary tool for individualized risk stratification upon admission, facilitating proactive clinical management.

## Data Availability

The datasets used in this study can be obtained from the corresponding author with a reasonable request. Requests to access these datasets should be directed to LR, renlinlin@qdu.edu.cn.
