# Supplementary material for: Predictive value of sarcopenia-related clinical indices for early disease progression in acute pancreatitis: a multicenter retrospective cohort study
Source: Front Nutr. 2026 Jul 23;13:1836073. doi: 10.3389/fnut.2026.1836073 (PMC13441785; doi:10.3389/fnut.2026.1836073)
Supplement: Supplementary file 1 [file Table_1.docx]

**Appendix A.1. Comparison between the sarcopenia group and the No-sarcopenia group**

| **Variables** | **No-sarcopenia (n = 356)** | **Sarcopenia (n = 114)** | ***P*** |
| --- | --- | --- | --- |
| Gender |  |  | 0.54 |
| Female | 232 (65.17) | 70 (61.40) |  |
| Male | 124 (34.83) | 44 (38.60) |  |
| Age, years |  |  | <0.001 |
| <45 | 167 (46.91) | 24 (21.05) |  |
| 45-65 | 106 (29.78) | 43 (37.72) |  |
| >65 | 83 (23.31) | 47 (41.23) |  |
| BMI, kg/m2 |  |  | <0.001 |
| <18 | 6 (1.69) | 5 (4.39) |  |
| 18-24 | 83 (23.31) | 50 (43.86) |  |
| 24-28 | 138 (38.76) | 46 (40.35) |  |
| >28 | 129 (36.24) | 13 (11.40) |  |
| Hypertension |  |  | 0.940 |
| No | 234 (65.73) | 76 (66.67) |  |
| Yes | 122 (34.27) | 38 (33.33) |  |
| Diabetes |  |  | 0.210 |
| No | 261 (73.31) | 76 (66.67) |  |
| Yes | 95 (26.69) | 38 (33.33) |  |
| Etiology |  |  | <0.001 |
| Gallstone-related | 126 (35.39) | 56 (49.12) |  |
| Alcohol-related | 176 (49.44) | 36 (31.58) |  |
| Hyperlipidemia-related | 54 (15.17) | 22 (19.30) |  |
| Smoke |  |  | 0.510 |
| No | 254 (71.35) | 77 (67.54) |  |
| Yes | 102 (28.65) | 37 (32.46) |  |
| Drink |  |  | 0.380 |
| No | 245 (68.82) | 84 (73.68) |  |
| Yes | 111 (31.18) | 30 (26.32) |  |
| PMA, cm2 | 16.56 (12.71-21.79) | 8.12 (6.73-10.02) | <0.001 |
| PMI, cm2/m2 | 5.64 (4.50-7.28) | 2.88 (2.36-3.48) | <0.001 |
| PMD, HU | 43.00 (36.88-47.50) | 35.50 (30.50-43.50) | <0.001 |
| Erythrocyte, 109/L | 4.62 (3.95-5.23) | 4.29 (3.76-4.76) | <0.001 |
| Leukocytes, 109/L | 12.66 (8.89-16.73) | 10.68 (7.27-14.85) | 0.020 |
| Neutrophils, 109/L | 10.48 (6.67-14.37) | 8.86 (4.83-12.83) | 0.020 |
| Platelets, 109/L | 216.00 (169.75-280.00) | 205.00 (172.00-252.25) | 0.330 |
| Lymphocytes, 109/L | 1.14 (0.81-1.69) | 1.12 (0.81-1.56) | 0.830 |
| Hematocrit, % | 42.25 (36.50-46.80) | 39.60 (33.92-44.38) | <0.001 |
| PCT, ng/ml | 0.58 (0.12-1.85) | 0.56 (0.08-2.96) | 0.670 |
| CRP, mg/L | 85.89 (13.01-160.68) | 56.00 (12.21-157.76) | 0.360 |
| NLR | 8.83 (4.80-14.43) | 7.87 (3.78-15.26) | 0.250 |
| SII | 1943.74 (982.67-3119.17) | 1409.31 (718.57-3351.92) | 0.140 |
| ALT, U/L | 35.00 (21.80-53.00) | 35.61 (20.00-66.50) | 0.960 |
| AST, U/L | 32.20 (21.00-56.00) | 32.00 (19.89-78.10) | 0.680 |
| Alb, g/L | 35.91 (30.66-40.02) | 34.12 (28.85-41.31) | 0.080 |
| Urea nitrogen, mmol/L | 5.50 (3.80-7.30) | 5.92 (4.31-8.73) | 0.020 |
| Creatinine, umol/L | 65.45 (53.00-90.30) | 68.50 (52.08-87.92) | 0.920 |
| Total bilirubin, umol/L | 18.32 (13.57-25.02) | 15.76 (11.14-24.76) | 0.080 |
| Glucose, mmol/L | 9.98 (7.44-13.60) | 8.64 (6.26-13.00) | 0.030 |
| serum calcium, mmol/L |  |  | 0.200 |
| 2.10-2.55 | 164 (46.07) | 44 (38.60) |  |
| ＜2.10, ＞2.55 | 192 (53.93) | 70 (61.40) |  |
| serum sodium, mmol/L |  |  | 0.940 |
| 135-145 | 247 (69.38) | 78 (68.42) |  |
| ＜135, ＞145 | 109 (30.62) | 36 (31.58) |  |
| Revised Atlanta classification |  |  | 0.010 |
| Mild | 171 (48.03) | 37 (32.46) |  |
| Moderately severe | 66 (18.54) | 23 (20.18) |  |
| Severe | 119 (33.43) | 54 (47.37) |  |
| Intra-abdominal fluid collection |  |  | 0.230 |
| No | 215 (60.39%) | 61 (53.51%) |  |
| Yes | 141 (39.61%) | 53 (46.49%) |  |
| Pancreatic pseudocyst |  |  | 0.080 |
| No | 330 (92.70%) | 99 (86.84%) |  |
| Yes | 26 (7.30%) | 15 (13.16%) |  |
| Walled-off necrosis |  |  | 0.010 |
| No | 316 (88.76%) | 89 (78.07%) |  |
| Yes | 40 (11.24%) | 25 (21.93%) |  |
| SIRS |  |  | 0.130 |
| No | 315 (88.48%) | 94 (82.46%) |  |
| Yes | 41 (11.52%) | 20 (17.54%) |  |
| ARDS |  |  | 0.090 |
| No | 274 (76.97%) | 78 (68.42%) |  |
| Yes | 82 (23.03%) | 36 (31.58%) |  |
| Mortality |  |  | 0.030 |
| No | 348 (97.75%) | 106 (92.98%) |  |
| Yes | 8 (2.25%) | 8 (7.02%) |  |
| LOS, days | 17.00 (10.00-28.25) | 16.50 (10.00-27.00) | 0.990 |

Sarcopenia grouping is based on gender-specific L3-PMI cut-off values (male: <5.41 cm²/m²; female: <3.32 cm²/m²).
